# Omnidirectional tele-perception enabled by nano-architectured electret skin

**DOI:** 10.1016/j.isci.2025.114584

**Published:** 2025-12-31

**Authors:** Yan Du, Zhiwei Zhang, Zhong Lin Wang, Di Wei

**Affiliations:** 1 Beijing Institute of Nanoenergy and Nanosystems, Chinese Academy of Sciences, Beijing 101400, China; 2 School of Nanoscience and Engineering, University of Chinese Academy of Sciences, Beijing 100049, China

**Keywords:** Biotechnology, Applied sciences

## Abstract

The environmental perception capability of embodied intelligent systems is highly dependent on their physical interactions with the surrounding environment, where tele-perception serves as a key technology enabling adaptive interaction and real-time human-machine interaction (HMI). However, existing tele-perception systems are fundamentally constrained by their underlying physical mechanisms and environmental disturbances, resulting in limited sensing directionality, poor spatial resolution, and inadequate environmental robustness. To address these challenges, this study develops an omnidirectional nano-architectured electret skin (NAES) by precisely tuning charge-trapping units within the established heterogeneous interface of the charge transport layer (CTL) and charge blocking layer (CBL). The proposed architecture arranges NAES units along 0°, 45°, 90°, 135°, and four diagonal orientations, leveraging the anisotropic electrostatic disturbance responses of each unit to achieve high-precision tele-perception of omnidirectional targets in three-dimensional space. This design overcomes the unidirectional sensing limitation of conventional NAES systems, enabling enhanced spatial perception and adaptive omnidirectional interaction in complex, dynamic environments.

## Highlights

• Eight NAES units enable omnidirectional electrostatic tele-perception
• CTL–CBL charge trapping ensures stable and high-fidelity electrostatic sensing
• Directional signal patterns allow accurate target orientation recognition
• The design overcomes unidirectional limits in HMI applications

## Graphical abstract

**Figure:**
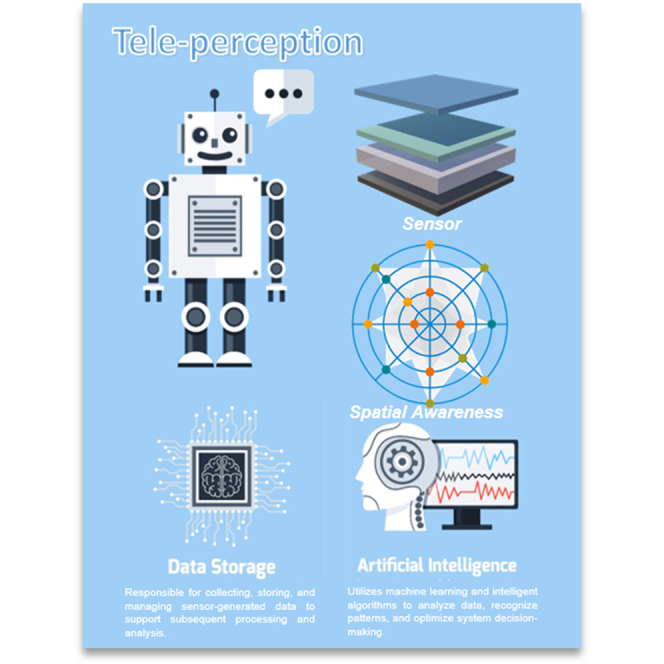

## Introduction

Embodied intelligence theory posits that a machine’s capability to perceive targets critically depends on its interactions with the physical environment, with tele-perception serving as a core technology for enabling adaptive interaction and real-time human-machine interaction (HMI).^1^^,^^2^ Tele-perception directly drives technological innovation in autonomous robots,^3^^,^^4^^,^^5^ intelligent wearable devices,^6^^,^^7^ and related fields. Compared with traditional passive sensing paradigms, tele-perception achieves active environmental cognition through spatiotemporal coupling of charge fields, endowing machines with behavioral plasticity and interaction autonomy in dynamic scenarios. It thus represents a key approach to overcoming the “physical contact dependence” bottleneck in conventional HMI.^8^^,^^9^^,^^10^^,^^11^ However, mainstream tele-perception technologies are fundamentally constrained by their physical principles and commonly exhibit limited environmental adaptability, sensing range, and modality-specific blind spots. Infrared sensors are susceptible to humidity-induced signal attenuation,^12^^,^^13^ ultrasonic sensors suffer from wave scattering in complex environments,^14^^,^^15^^,^^16^ laser sensors are affected by airborne particulates,^17^^,^^18^ and capacitive,^19^^,^^20^^,^^21^ magnetic,^22^^,^^23^ and optical^24^^,^^25^^,^^26^ sensors are, respectively, limited by temperature and humidity fluctuations, electromagnetic interference, and line-of-sight occlusion. These limitations severely restrict the deployment of embodied intelligent systems in interference-rich and dynamically changing scenarios.

To address the inherent limitations of conventional sensing technologies, in 2024, Wei et al. proposed a bio-inspired electrosensory tele-perception system,^27^^,^^28^ which mimics the electroreception mechanism of the platypus to construct stable charge traps as electroreceptors. This system achieved a sensing distance of 1.55 m for human targets and a sensitivity of ΔV/Δd = 14.2, thereby significantly exceeding conventional sensing benchmarks. By integrating multimodal data fusion with machine learning algorithms,^29^^,^^30^ the system effectively filtered environmental noise, resolved signal ambiguities, and leveraged electrostatic disturbance sensing to eliminate line-of-sight dependency, thereby overcoming the environmental vulnerabilities of both infrared and ultrasonic techniques. Building on this foundation, Wei et al., in 2025, further optimized the design of the heterogeneous meta-structure interface and proposed a nano-architectured electret skin (NAES).^31^ By leveraging the synergistic effects of a charge transport layer (CTL) and a charge blocking layer (CBL), NAES realized a critical transition from surface-confined charge retention to volumetric charge trapping, extending the sensing distance to 3 m and enhancing sensitivity to ΔV/Δd = 21.8, while also improving robustness under high humidity and temperature fluctuations. Despite these advances in sensing distance, sensitivity, and environmental adaptability, existing NAES systems remain limited to unidirectional tele-perception, incapable of simultaneous detection and orientation discrimination of omnidirectional targets in three-dimensional space. This limitation significantly restricts their applications in autonomous robot obstacle avoidance, multi-target tracking, and complex HMI, forming an advanced bottleneck for the spatial perception capabilities of embodied intelligent systems.

Herein, the present work extends the research by controlling the spatial arrangement of NAES units within the established heterogeneous interface of the CTL and the CBL to construct an eight-directional NAES architecture. NAES units are arranged along 0°, 45°, 90°, 135°, and four diagonal directions, and the anisotropic responses of each unit to electrostatic disturbances enable high-precision tele-perception of omnidirectional targets in three-dimensional space. This study not only overcomes the unidirectional sensing limitation of conventional NAES systems but also relies on the established bulk charge-trapping mechanism (CTL-mediated dynamic charge redistribution and SrTiO_3_ nanoparticle polarization in the CBL for charge stabilization). Furthermore, analysis of the directional features of signals from the eight units allows accurate determination of target orientation. This architecture endows embodied intelligent systems with more comprehensive spatial cognition, effectively filling the gap in unidirectional sensing capabilities in previous NAES designs and further promotes their practical application in autonomous robot omnidirectional obstacle avoidance and complex HMI.

## Results

### Design of NAES for omnidirectional tele-perception

The environmental perception capability of embodied intelligent systems directly determines their behavioral autonomy and interaction efficiency in dynamic scenarios. Conventional sensing systems are typically constrained by unidirectional detection, line-of-sight dependence, and environmental fragility, making it difficult to simultaneously monitor multiple targets across different directions. To overcome these limitations, this study proposes an omnidirectional tele-perception strategy based on NAES, which employs spatially distributed units to actively detect targets in three-dimensional space (Figures S1 and S2). Each NAES unit consists of three key components: the CTL, the CBL, and the Ni-metal mesh cloth electrode. The CTL enables dynamic charge redistribution, the CBL stabilizes trapped charges, and the electrode converts electrostatic interactions into measurable signals, allowing high-sensitivity omnidirectional tele-perception.

This architecture not only enables simultaneous sensing of electrostatic disturbances from multiple directions but also allows extraction of directional information, thereby providing embodied intelligent platforms with enhanced spatial cognition.

**Figure 1:**
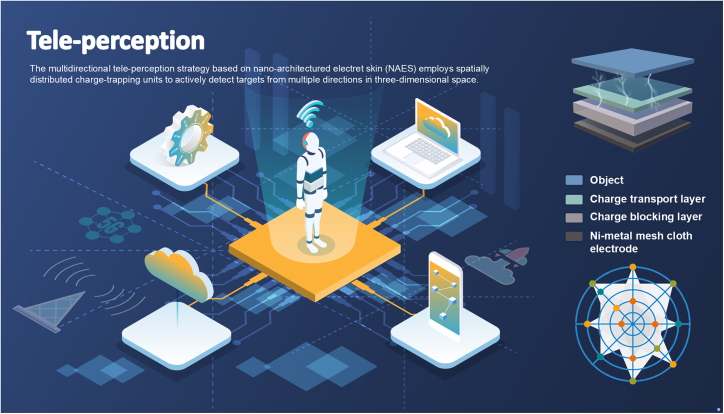
Omnidirectional tele-perception in embodied intelligent systems enabled by NAES.

The conceptual framework of NAES for omnidirectional tele-perception in embodied intelligent systems is illustrated in Figure 1. NAES architecture consists of spatially distributed NAES units arranged along specific orientations, which enable active detection of electrostatic field perturbations from multiple directions and achieve precise three-dimensional target localization. Each directional unit exhibits anisotropic responses to external electrostatic disturbances, allowing the system to concurrently extract omnidirectional electric field information and facilitate target orientation discrimination. In contrast to conventional NAES systems with unidirectional sensing, this omnidirectional layout significantly enhances spatial perception, enabling simultaneous monitoring of multiple targets in dynamic environments and expanding the perceptual capabilities of embodied intelligent platforms. By analyzing the directional output signals, NAES achieves precise target orientation discrimination, providing embodied intelligent systems with comprehensive three-dimensional spatial cognition.

This design not only demonstrates the synergistic principle of omnidirectional charge trapping and tele-perception but also offers a practical sensing solution for autonomous robotic navigation, omnidirectional obstacle avoidance, complex HMI, and intelligent interactions in dynamic environments. By integrating an omnidirectional layout with established charge-trapping mechanisms, the NAES architecture provides an advanced technological pathway and implementation framework for high-dimensional spatial perception in embodied intelligent systems, laying a solid foundation for subsequent experimental validation and performance evaluation.

### Mechanism and simulation of omnidirectional tele-perception in NAES

**Figure 2:**
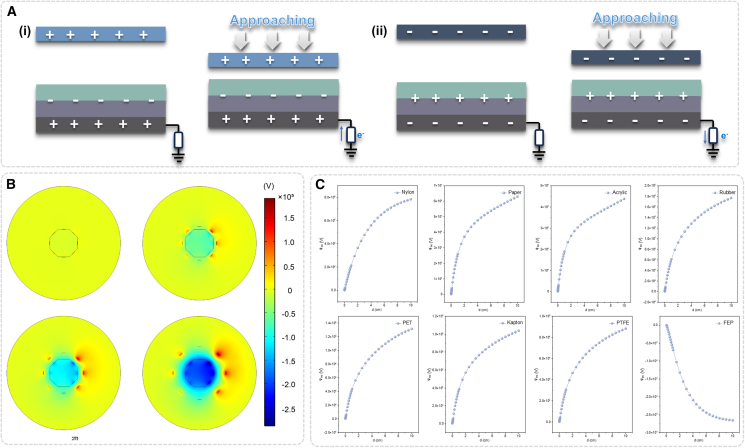
Working principle and electrostatic field simulation of NAES omnidirectional tele-perception (A) Mechanism of omnidirectional tele-perception in NAES: (i) positively charged and (ii) negatively charged. (B) COMSOL-simulated potential distribution of NAES under different states for omnidirectional tele-perception. (C) Potential distribution of different NAES materials at various distances for omnidirectional tele-perception.

The omnidirectional tele-perception principle of NAES under positively and negatively charged states is illustrated in Figure 2A. Each charge-trapping unit responds to directional variations in external electrostatic field disturbances and extracts target orientation information through differential potential changes, thereby enabling directional discrimination in three-dimensional space. This mechanism takes full advantage of the anisotropic response characteristics of NAES units, allowing the system to maintain high sensitivity and precise localization capability in omnidirectional and multi-target environments. Furthermore, the simulated potential distribution of NAES obtained by COMSOL is presented in Figure 2B. The omnidirectional units exhibited distinct potential gradients and spatial distribution features, clearly revealing their response patterns and effective sensing ranges under external electrostatic perturbations. The simulation results validate the cooperative sensing behavior among the omnidirectional units within the designed architecture, providing theoretical support for spatial localization and orientation discrimination, as well as a visual foundation for optimizing omnidirectional configurations.

In addition, the potential distribution characteristics of NAES fabricated from different materials at varying distances are shown in Figure 2C. The results indicate that material selection significantly influences sensing sensitivity, potential amplitude, and effective detection distance. The electrode potential distributions of NAES units fabricated from different triboelectric materials at varying distances from the target are presented in Figure S3. These findings offer essential guidance for optimizing NAES architecture and the spatial arrangement of omnidirectional units, enabling high-precision tele-perception in environments with diverse materials and multiple targets.

Overall, these analyses not only provide visual validation for the system design but also establish a solid theoretical basis for enhancing omnidirectional configuration, sensing sensitivity, and environmental adaptability. The mechanistic insights into NAES-based omnidirectional tele-perception lay the foundation for achieving high-dimensional spatial cognition and precise long-range interaction in embodied intelligent systems under complex dynamic scenarios.

### Output characteristics of NAES for omnidirectional tele-perception

**Figure 3:**
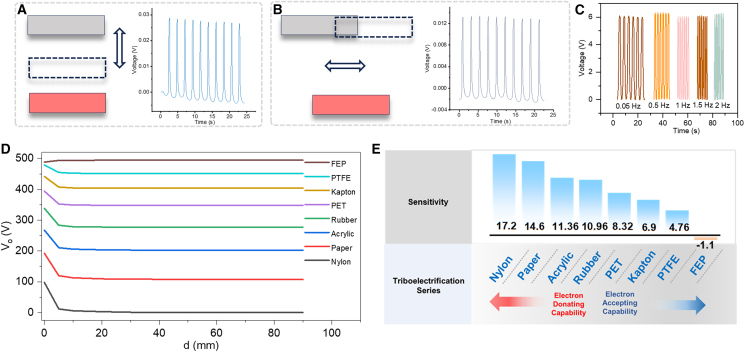
Performance evaluation of NAES in omnidirectional tele-perception (A) Longitudinal tele-perception of NAES. (B) Lateral sliding tele-perception of NAES. (C) Output voltage of NAES under different frequencies. (D) Output voltage of NAES with different materials at various distances. (E) Sensitivity of NAES for different materials.

To evaluate the output performance of NAES in omnidirectional tele-perception, its longitudinal, lateral, and frequency-dependent characteristics were systematically investigated. As shown in Figure 3A, when a nylon target approached vertically within a range of 100 mm, NAES generated stable and reproducible voltage responses to the target displacement, indicating excellent spatial resolution and signal stability. Figure 3B further demonstrates the lateral sensing performance of NAES, where the system accurately responds to the horizontal motion trajectory of the target, enabling precise tracking and recognition of dynamic objects from multiple directions. The output characteristics of NAES under different excitation frequencies are presented in Figure 3C. When the nylon target is positioned at a distance of 10 mm, the output voltage remains stable with increasing frequency, demonstrating superior frequency adaptability and response robustness. The output voltage distributions of NAES fabricated with different materials at various distances are shown in Figure 3D. As the target moves farther away, the intensity of the induced electrostatic field at the sensor surface diminishes, leading to lower charge transfer in the CTL-CBL structure and a corresponding decrease in measured voltage. The variation in voltage amplitude among different materials reflects the significant influence of material properties on the sensing sensitivity and detection range of the system. Figure 3E further quantifies the sensitivity differences across materials, highlighting the critical role of material engineering in optimizing the sensing performance of NAES.

To highlight the advantages of the proposed NAES system, we compared it with several recent omnidirectional sensing devices^32^^,^^33^^,^^34^^,^^35^ (Table S1). Unlike purely mechanical or capacitive proximity sensors, NAES enables non-contact, long-range electrostatic sensing. Its spatially distributed array provides directional discrimination, allowing determination of target orientation in addition to distance. By incorporating different triboelectric materials, NAES exhibits material-dependent responses, enhancing target recognition capabilities. Furthermore, the CTL-CBL structure with charge stabilization improves robustness under varying environmental conditions, making NAES well suited for multidimensional tele-perception in dynamic scenarios.

In summary, NAES exhibits high sensitivity and stable output characteristics under omnidirectional and multi-frequency conditions, enabling reliable identification of targets with different orientations and motion states. These results provide advanced design insights and experimental foundations for developing high-resolution and robust embodied intelligent sensing platforms.

### Machine learning-enabled omnidirectional tele-perception

**Figure 4:**
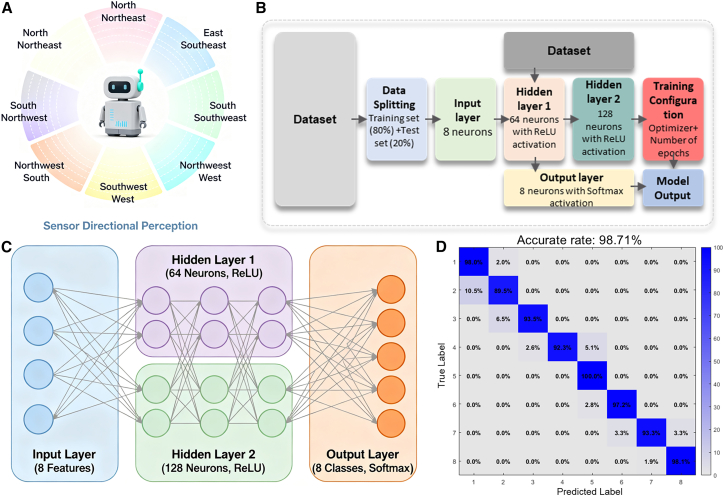
Machine learning analysis of NAES omnidirectional tele-perception (A) Schematic illustration of omnidirectional tele-perception by NAES. (B) Overall computational flowchart of the machine learning process. (C) Architecture of the multilayer perceptron (MLP) model. (D) Confusion matrix of the machine learning results.

The schematic illustration of NAES omnidirectional tele-perception is shown in Figure 4A. To enhance the data interpretation capability of NAES for omnidirectional tele-perception, a multilayer perceptron (MLP) is employed to perform classification on a multi-class tabular dataset. The dataset is self-generated, containing 8 feature dimensions and 8 classes, with 200 samples per class, totaling 1600 samples. The classes are well separated in the feature space, and Gaussian noise is added to simulate variations in real-world data distributions. All features are standardized and randomly split into training and test sets, with the test set accounting for 20% of the total data.

The overall machine learning workflow is presented in Figure 4B, including data preprocessing, training-test split, model training, and prediction evaluation, providing a systematic computational pipeline for decoding omnidirectional signals. The constructed MLP network architecture is shown in Figure 4C, which includes an input layer of 8 neurons, two hidden layers with 64 and 128 neurons (ReLU activation), and an output layer of 8 neurons (Softmax activation). The model is trained for 100 epochs using the Adam optimizer. The experimental results indicate that the model achieves an average classification accuracy of 98.71% on the test set, demonstrating excellent performance (Figure 4D).

This approach not only validates the distinguishability of omnidirectional NAES signals but also provides a reliable algorithmic basis for target orientation discrimination, supporting high-dimensional spatial perception and decision-making in embodied intelligent systems operating in complex dynamic environments.

### Simulated applications of NAES-based omnidirectional tele-perception

**Figure 5:**
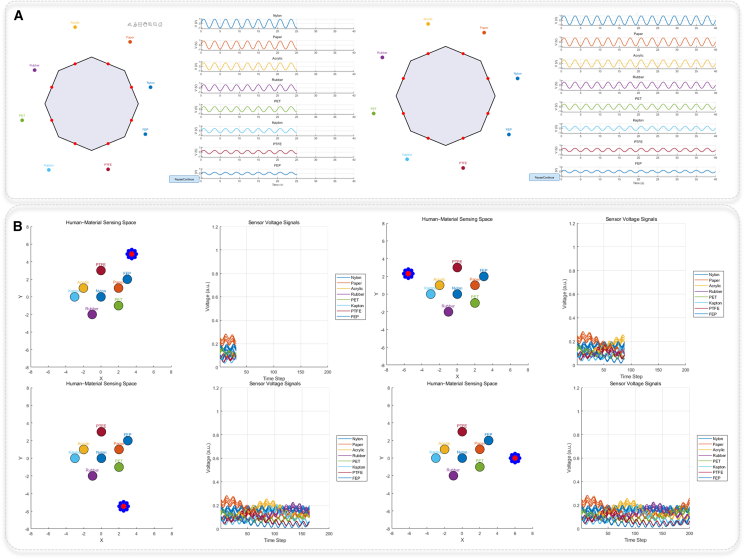
Simulated applications of NAES for omnidirectional tele-perception (A) Eight-directional NAES architecture demonstrating tele-perception of approaching targets, with each direction exposed to a distinct material. (B) Moving multi-array NAES units performing spatial tele-perception of multiple surrounding materials.

To evaluate the applicability of NAES in dynamic scenarios, a MATLAB-based dynamic visualization simulation of the octagonal NAES architecture was conducted. This simulation framework allowed controlled analysis of the spatial sensing behavior in omnidirectional and multi-material conditions, enabling direct observation of how each unit responds to electrostatic disturbances in real time. By reproducing the voltage characteristics of different triboelectric materials and mapping the corresponding spatial potential distribution, the simulation provided comprehensive insight into the directional differentiation and multi-point sensing mechanisms of the NAES system. Such a simulation-based approach is well suited for revealing the spatial response patterns of the array, making it an effective method for assessing its sensing performance. The simulation model is based on a regular octagon, with an independent NAES sensor unit placed at the midpoint of each edge. The spatial configuration of the NAES array is designed to achieve omnidirectional tele-perception, with units arranged along 0°, 45°, 90°, 135°, and four diagonal orientations. This arrangement is chosen to ensure comprehensive directional coverage, minimize blind spots, and enable precise detection of targets approaching from any angle. Each unit corresponds to a different triboelectric material (nylon, paper, acrylic, rubber, polyethylene terephthalate (PET), kapton, polytetrafluoroethylene (PTFE), fluorinated ethylene propylene (FEP)) and acts as an independent sensing module, detecting charge transfer induced by relative motion and generating alternating voltage signals (Figure 5A and Note S1). By placing the sensors at these orientations, the system can actively sense targets from multiple directions simultaneously, improving spatial resolution, enabling multi-target tracking, and enhancing the overall perception capabilities of the embodied intelligent system.

Furthermore, the model simulates a circular sensor array moving along a central circular trajectory representing a “human body,” demonstrating the dynamic response of the multi-channel sensors to surrounding stationary material targets (Figures 5B and Note S2). The simulation environment is a two-dimensional plane, with the central red dot representing the moving platform carrying the sensors, and eight blue sensor nodes evenly distributed around it to form a circular array with a radius of approximately 0.5 units. The moving platform follows a circular trajectory with radius R = 6, causing the relative distances between each sensor and the material targets to vary over time and producing temporally sequenced multi-channel voltage responses. The eight stationary triboelectric material targets represent different surface charge transfer properties and triboelectric potentials, which were reflected by varying voltage amplitude coefficients, enabling dynamic perception of multi-material and omnidirectional targets. We analyze the simulation data to determine the minimum detectable distance and response time of each NAES unit. The response threshold is defined as the smallest target displacement producing a voltage above 0.05 V and the response time as the interval until this threshold is reached. As summarized in Table S2, the NAES units exhibit rapid response, with response times on the order of milliseconds, demonstrating reliable detection under realistic conditions. To evaluate the robustness of the NAES under realistic conditions, we simulate environmental disturbances by introducing both Gaussian and uniform noise into the sensor signals. The effects of noise are quantified in terms of signal fluctuation and detection reliability. As summarized in Table S3, the NAES units maintain high detection reliability (95%–98%) and limited signal fluctuations (±5%–7%) under these conditions, demonstrating stable and reliable performance in the presence of environmental interference.

Overall, this simulation provides an intuitive demonstration of the working principles of NAES for omnidirectional tele-perception, validates the effects of multi-channel arrangement, circular layout, and material heterogeneity on spatial sensing performance, and offers theoretical and visual guidance for the design of embodied intelligent systems in complex dynamic environments.

## Discussion

In summary, this study systematically constructed and validated an omnidirectional tele-perception system based on NAES. By designing a spatial layout of eight-directional NAES units, NAES not only overcame the limitations of conventional unidirectional tele-perception, achieving simultaneous detection and orientation discrimination of targets in three-dimensional space, but also relied on the established bulk charge-trapping mechanism. Experimental results demonstrated that NAES exhibited stable and high-precision output characteristics in longitudinal, lateral, and frequency-dependent sensing. Multi-material experiments further confirmed the significant impact of material selection on sensitivity and sensing range. By employing an MLP for omnidirectional signal classification, precise target orientation discrimination was achieved, confirming the distinguishability of the omnidirectional output signals and providing a reliable algorithmic foundation for high-dimensional spatial perception in complex dynamic scenarios. Simulation studies showed that a multi-channel ring array combined with different materials could effectively achieve dynamic sensing of moving targets and static multi-material environments, providing visual validation and optimization guidance for embodied intelligent systems in practical applications. Overall, this work realized omnidirectional tele-perception based on NAES, systematically validating its feasibility for spatial localization, target recognition, and multi-material environments, and offering an advanced sensing design strategy and technological framework for autonomous robotic navigation, complex HMI, and dynamic interactive scenarios in embodied intelligent systems.

## Resource availability

### Lead contact

Requests for further information and resources should be directed to and will be fulfilled by the lead contact, Di Wei (weidi@binn.cas.cn).

### Materials availability

The materials used in this study are available from the corresponding authors upon reasonable request.

### Data and code availability

• All data reported in this paper will be shared by the lead contact upon request.
• This paper does not report original code.
• Any additional information required to reanalyze the data reported in this paper is available from the lead contact upon request

## Acknowledgments

This work was supported by the 10.13039/100014718National Natural Science Foundation (grant no. 22479016).

## Author contributions

D.W. and Z.L.W. proposed the idea and the project. D.W. designed all the experiments and supervised the whole project. Y.D. carried out the experiments in this paper. Z.W.Z. provided support for theoretical simulations. All the authors discussed the results and commented on the manuscript. D.W. and Y.D. wrote this paper.

## Declaration of interests

The authors declare no conflict of interest.

## STAR★Methods

### Key resources table

**Table:** 

REAGENT or RESOURCE	SOURCE	IDENTIFIER
**Chemicals, peptides, and recombinant proteins**		

Silicon wafers	Aladdin (Shanghai, China)	CAS: 7782-61-8
PDMS (Polydimethylsiloxane)	Aladdin (Shanghai, China)	CAS: 63148-62-9
Curing agent	Aladdin (Shanghai, China)	CAS: 63148-62-9
SrTiO_3_ (Strontium Titanate) powders	Aladdin (Shanghai, China)	CAS: 12060-59-2
Carbon black powder	Aladdin (Shanghai, China)	CAS: 1333-86-4

### Experimental model and subject participant details

This study does not use experimental methods typical in the life sciences.

### Method details

#### Manufacture of NAES

NAES based on structured nanocomposites was successfully fabricated by bonding a plasma treated carbon black doped polydimethylsiloxane (PDMS) film, a PDMS film structured with strontium titanate (SrTiO_3_) nanoparticle doping, and the Ni-metal mesh. The detailed fabrication procedures, component ratios, and step-by-step experimental protocols for NAES are provided in our previous publication (Matter, 8, 102363, 2025, https://doi.org/10.1016/j.matt.2025.102363).

#### Electric measurement and characterization

A linear motor (LinMotS01-72/500) was employed to control the distance between objects and NAES.Plasma cleaner provided voltage for the pre-charging process. Open circuit voltage was measured using a programmable electrostatic voltmeter (Keithley 6514). For basic output performance testing of NAES, a programmable electrostatic voltmeter (Keithley 6514) was directly connected to a synchronized data acquisition card (National Instruments 6346) to measure multi-channel voltage signals.

### Quantification and statistical analysis

Figures represent averaged or representative results of multiple independent experiments. Analyses and plots were performed with Origin.

### Additional resources

This study has not generated or contributed to a new website/forum.

## Supplemental information

Document S1. Figures S1–S3 and Tables S1–S3, and Notes S1–S2
