# Supplementary material for: Omnidirectional tele-perception enabled by nano-architectured electret skin
Source: iScience. 2025 Dec 31;29(2):114584. doi: 10.1016/j.isci.2025.114584 (PMC12855575; doi:10.1016/j.isci.2025.114584)
Supplement: Document S1. Figures S1–S3 and Tables S1–S3, and Notes S1–S2 [file mmc1.pdf]

iScience, Volume *29*

Supplemental information

Omnidirectional tele-perception enabled by nano-architectured electret skin

Yan Du, Zhiwei Zhang, Zhong Lin Wang, and Di Wei

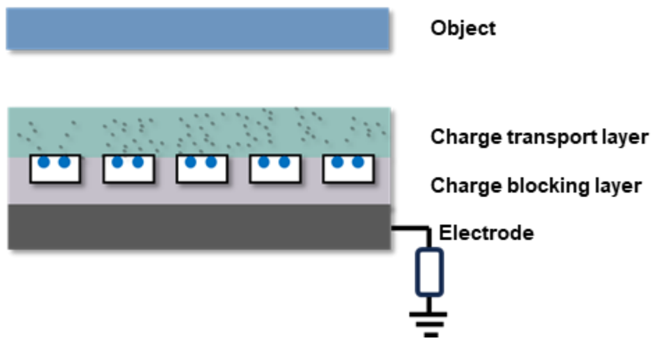

Figure S1. Schematic diagram of the NAES structure.

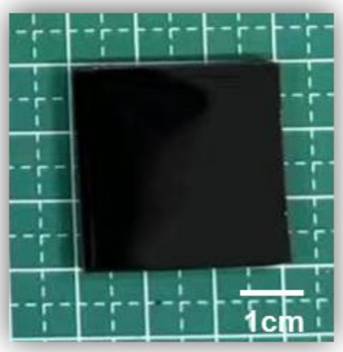

Figure S2. Photograph of the NAES.

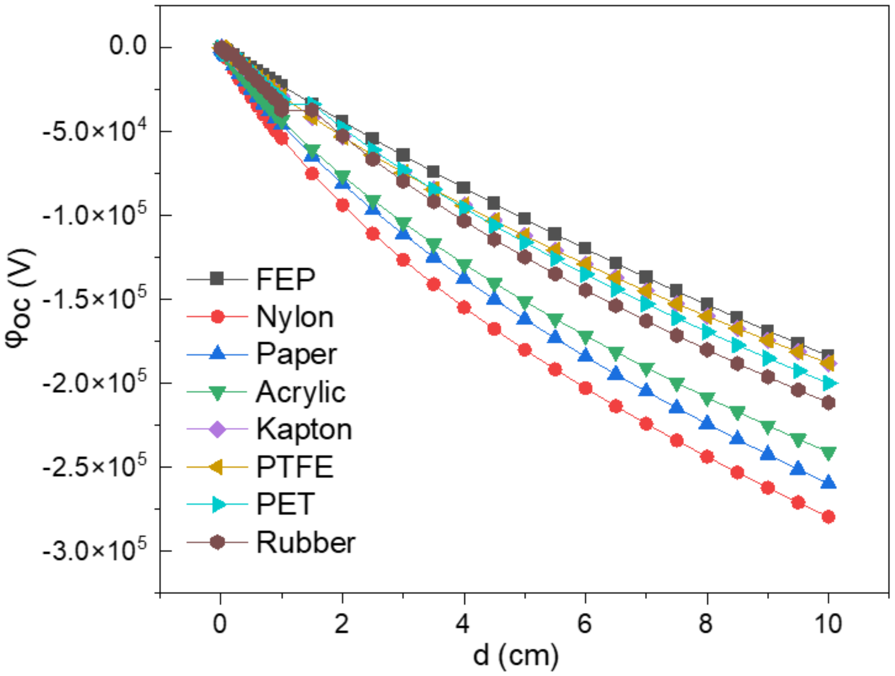

Figure S3. Electrode potential distribution of different NAES materials at varying distances for multidirectional tele-perception.

Note S1 Simulation principles.

Each sensing node generates an alternating voltage signal based on sinusoidal motion of the contacting surface:

where:

$$V_i(t) = A_i \cdot \sin(2\pi f_i t)$$

- $V_i(t)$ —instantaneous voltage output of material *i*
- A_i —normalized amplitude representing charge generation capability
- f_i —excitation frequency corresponding to contact motion
- t— time (s)

The motion of the interacting object is simulated as a harmonic variation in distance:

$$d_i(t) = d_0 + \Delta d \cdot \sin(2\pi f_i t)$$

This distance controls both the visual position of each sensor marker and indirectly the amplitude of the corresponding electrical signal.

Note S2 Signal computation.

The voltage signal at each sensor due to each material is computed conceptually as:

$$V_{s,o}(t) = A_o \cdot \frac{1}{d_{s,o}(t) + \epsilon} + \eta_{s,o}(t)$$

Where:

- $V_{s,o}(t)$is the instantaneous voltage at sensor *s* from material *o*
- $A_o$is the material-dependent amplitude coefficient
- $d_{s,o}(t)$is the distance between sensor *s* and material *o* at time *t*
- ϵprevents division by zero
- $\eta_{s,o}(t)$represents small simulated noise

This formula is described in words only; no code is disclosed.

Table S1. Comparison of NAES with omnidirectional sensing devices.

Sensing Mode	Key Performance	Comparison to Our NAES Work	Reference
Hall-effect pressure sensor	Sensitivity ~ 61.34 mV/kPa over 4–90 kPa	This is quite effective for contact pressure sensing, but it measures force only, not electrostatic fields, and does not support long-range tele-perception. Our NAES system, by contrast, detects electrostatic disturbances, enabling non-contact sensing at a distance.	32
Capacitive proximity sensor	Sensing range of 5–10 cm, very low power (≈ 5 mW)	This device demonstrates good omnidirectional proximity detection with power efficiency. However, its capacitive principle primarily senses distance, without directional discrimination of electrostatic field vectors. Our NAES array provides directional orientation resolution in addition to distance, enhancing spatial cognition.	33
Capacitive tactile sensor	Sensitivity ≈ 0.306 kPa^{-1}, response range 2.55 Pa to 160 kPa, > 99% angle recognition	This e-skin achieves high sensitivity and accurate angular force discrimination, but it requires physical contact. In contrast, our NAES implementation supports non-contact, omnidirectional electrostatic tele-perception, enabling more flexible interaction and longer-range sensing.	34
Triboelectric whisker array	Reconfigurable array for human–machine–environment interaction	Their system provides interesting multi-directional mechanical interaction via “whiskers,” but its sensing is based on mechanical deformation, not on remote electrostatic field detection. The NAES system uniquely combines charge-trapping layers to sense fields without requiring physical contact or whisker-like structures.	35

Table S2. Material and signal parameters.

Index	Material	Amplitude (V)	Frequency (Hz)	Response Threshold(m)	Physical Note
1	**Nylon**	1.0	0.35	0.16	Strong triboelectric activity, highest output
2	**Paper**	0.9	0.40	0.14	High surface charge density
3	**Acrylic**	0.8	0.45	0.13	Stable dielectric behavior
4	**Rubber**	0.7	0.50	0.11	Flexible and resilient
5	**PET**	0.6	0.20	0.08	Common polymer, moderate output
6	**Kapton**	0.5	0.25	0.07	High temperature resistance
7	**PTFE**	0.4	0.30	0.06	Electronegative surface, lower effective output
8	**FEP**	0.3	0.55	0.04	Weakest signal amplitude, high frequency

Table S3. Effect of environmental noise on NAES signal performance.

Material	Noise Type	Noise Amplitude (V)	Signal Fluctuation (%)	Detection Reliability (%)
**Nylon**	Gaussian	0.05	±5	98
**Paper**	Gaussian	0.05	±6	97
**Acrylic**	Uniform	0.05	±7	96
**Rubber**	Uniform	0.05	±6	95
**PET**	Gaussian	0.05	±5	98
**Kapton**	Gaussian	0.05	±6	97
**PTFE**	Uniform	0.05	±7	95
**FEP**	Uniform	0.05	±6	96
